# Effect of Mechanical Stimuli and Zoledronic Acid on the Femoral Bone Morphology in Rats with Obesity and Limited Mobility

**DOI:** 10.3390/jcm12010043

**Published:** 2022-12-21

**Authors:** Nazar M. Kostyshyn, Siemowit Muszyński, Ewa Tomaszewska, Agnieszka Tomczyk-Warunek, Iwona Puzio, Piotr Dobrowolski

**Affiliations:** 1 Department of Normal Physiology, Danylo Halytsky Lviv National Medical University, 79010 Lviv, Ukraine; 2 Department of Biophysics, Faculty of Environmental Biology, University of Life Sciences in Lublin, 20-950 Lublin, Poland; 3 Department of Animal Physiology, Faculty of Veterinary Medicine, University of Life Sciences in Lublin, Akademicka St. 12, 20-950 Lublin, Poland; 4 Locomotor System Research Department, Department of Rehabilitation and Physiotherapy, Medical University in Lublin, 20-090 Lublin, Poland; 5 Department of Functional Anatomy and Cytobiology, Maria Curie-Skłodowska University, 20-033 Lublin, Poland

**Keywords:** osteoporosis, rat model, whole-body vibration, zoledronic acid

## Abstract

Our study aimed to compare the impact of zoledronic acid and whole-body vibration (WBV) as a non-pharmacological method of treatment for early obesity/immobility-related osteoporosis in male rat models. In total, 36 male Wistar rats were assigned to the following groups: obese control with immobility (Control, *n* = 12) and two experimental groups (*n* = 12 each), including obese and immobile rats subjected to whole-body vibration with an acceleration level of 3 m/s^2^ g (obesity and immobility + WBV) and obese and immobile rats that received an intramuscular injection of zoledronic acid at a dose of 0.025 mg/kg (obesity and immobility + ZOL). After the 8th and 16th week of treatment, *n* = 6 rats from each group were euthanized and isolated femora were subjected to a histological examination of bone, and analysis of the expression of osteoprotegerin (OPG) and the receptor activator of nuclear factor kappa-B ligand (RANKL) involved in bone turnover and the amount of thin collagen fibers (PSR stain). The obtained results showed that short-term vibrotherapy (up to 8 weeks) can lead to improvement in bone remodeling in rat models with obesity and limited mobility.

## 1. Introduction

Nowadays, obesity has become significantly prevalent throughout the world, in particular due to lifestyle changes. This, in turn, leads to social and medical problems, increasing the risk of metabolic diseases, internal organ dysfunction, and skeletal pathology [1,2]. While the reduction of excess weight remains the primary pathogenic treatment for obese individuals, complex metabolic abnormalities, lifestyle disturbances, comorbid chronic diseases, as well as medication impact can themselves compromise the ability to preserve mineral mass and normal bone architecture [1,2,3,4,5]. Thus, a close connection between osteoporosis, a sedentary lifestyle, and obesity is observed [6,7]. These pathological diseases of the musculoskeletal system share common risk factors and are directly related to the fragility of bones and fractures.

The interaction of skeletal muscles and bones occurs through biomechanical signals that directly stimulate both tissues [8,9,10]. However, the impact of obesity at a young and mature age, as well as an increased load on the skeleton, requires further study since there is an inverse relationship between an increase in body mass index and bone mineral density [2,3,4,5,6,7]. In obese people, movement control gradually deteriorates and eventually leads to disability and immobility. Conversely, mechanical loading has a positive effect on bone mineral density (BMD) in adults, while stimulating bone remodeling [11,12,13].

Studies show examples of high-calorie diet and/or sugar use as one of the factors contributing to obesity [14,15]. Similarly, a sedentary lifestyle also leads to obesity. A number of studies indicate a change in the structural properties of bones caused by diet and a sedentary lifestyle, both in experimental animals and in humans. Along with a decrease in BMD, bone strength decreases and adverse microarchitectural changes in bone, most often occurring in the trabecular layer, progress over time [16,17]. Taking into consideration the above data, the study of bone mineral density is important for predicting the occurrence of osteoporotic fractures, and its mineral mass as a predictor of fracture risk. However, not only the study of the mineral component, which determines bone strength, but also the matrix proteins on which the crystalline component is layered, in particular hydroxyapatite, is important for assessing the condition [18].

Since bone is a mechanosensitive tissue, to correct remodeling and improve its condition, in addition to pharmacological drugs, it is suggested to use mechanical stimuli—physical exercises, which are effective in the prevention and treatment of osteoporosis. Although vigorous exercise increases bone density, few adults, especially those who are overweight, follow these exercise recommendations. Whole-body vibration (WBV) is a type of mechanical stimulation characterized by the transmission of mechanical vibration stimuli through a vibration platform [19]. Mechanical loading stimulates bone formation by reducing apoptosis and increasing the proliferation and differentiation of osteoblasts and osteocytes. This happens through the Wnt/β-Catenin intracellular signaling pathway and the Piezo-type mechanosensitive ion channel component 1 (Piezo1) protein [20,21,22]. Thus, the mechanical load provided by body weight is part of the hypothesis that has led to the widespread belief that obesity can prevent bone loss and osteoporosis. However, recent reports have shown that excess fat mass does not protect against osteoporosis and is associated with low bone mineral density. Clearly, the competing effects of obesity and mechanical stress on bone metabolism remain an active area of research, and a high-calorie/high-fat diet increases bone resorption and may negate any positive effects of mechanical stimuli on bone in particular [1,2,3,4,5,17]

In our study, the aim was to compare the effects of WBV and zoledronic acid (ZOL) on the structure and remodeling of femoral bone in rat models with obesity and limited mobility. We compared the research results with the data obtained under the action of zoledronic acid, a bisphosphonate that is often used in clinical practice. The selective effect of bisphosphonates on bone tissue is based on a high affinity for the mineralized component, and provides inhibition of osteoclast activity. Zoledronic acid has no adverse effect on the formation, mineralization, and mechanical properties of bone. In addition, zoledronic acid can enhance the differentiation of osteoblasts, increasing bone mineral density [23].

Since the use of zoledronic acid has certain limitations and contraindications, in particular gastrointestinal intolerance, nephrotoxicity, and even osteonecrosis [24,25], it was necessary to offer non-pharmacological methods that could complement the existing pharmacological schemes [25,26,27]. We do not have sufficient information on studies of skeletal status conditions of the combined exposure to a high-calorie diet with immobilization/sedentary lifestyle and effective methods of improvement or prevention. Therefore, our primary objective was to compare the trabecular structure and activity of bone cells in immobilized adolescent rats with induced obesity exposure to whole-body vibration, or treatment with bisphosphonate. Understanding the relationship between obesity and bone metabolism may help to identify new molecular targets that may enhance osteoblastogenesis while inhibiting adipogenesis and/or reducing osteoclastogenesis.

## 2. Materials and Methods

### 2.1. Animals and Experimental Design

The experiment was conducted on 36 2-month-old healthy male Wistar rats, weighing 180–200 g. The animals were kept under a 12 h light and dark cycle, an air temperature of 21–23 °C, and a relative humidity of 60 ± 10%. After a week of acclimatization, a high-caloric diet was started and the rats were kept in limited mobility conditions. The control group (Control, *n* = 12) consisted of rats subjected only to high-caloric diet and immobility, and in the experimental groups, whole-body vibration (WBV, *n* = 12) or zoledronic acid injection (ZOL, *n* = 12) were added to the control regimen.

For immobilization, the cage volume-reduction model of bed rest developed by Marmonti et al. [28,29] was used, in which cage space (2150, Tecniplast, Buguggiate (VA), Italy) was limited to 12 × 12 × 8 cm, which was 20% of the standard cage volume. The high-calorie obesifying “condensed milk” high-fat diet (D12266B, Research Diets, New Brunswick, NJ, USA) had an energy density of 4.41 kcal/g, with 51.4%, 31.8%, and 16.8% of energy coming from carbohydrates (of which 50% is from sucrose), fat, and protein, respectively, was used to induce obesity in rats [30,31,32,33,34,35].

The whole-body vibration (WBV) protocol was adapted from Xiao [36] with minor modifications [37]. Vertical vibration oscillations were modeled using a 250 W APC Rain-60 vibration pump (AquaPlanet Company, Ilfov, Romania) with a maximum pressure of 7 bar and an AFC-120 model voltage regulator (SophPower Electronics, Dongguan, China). A vibration platform with a container holding the experimental group of rats was attached to the stem of the vibration pump. The vibration acceleration level was 3.0 m/s^2^ (0.3 g) with a frequency of 50 Hz, which has been shown to stimulate trabecular bone formation rate in adolescents with clinical conditions and adults with low BMD or osteoporosis [38]. The rats were exposed to WBV continuously for 30 min (no breaks), 5 days a week, which was similar to the protocol proposed by Flieger et al. [39].

Zoledronic acid (ZOLTA^®®^, Amaxa Pharma, Lviv, Ukraine) at a dose of 0.025 mg/kg body weight was administered by intramuscular injection (quadriceps femoris) every 4 weeks. This route of administration was chosen due to the technical availability and slow distribution of the drug [37]. Groups not receiving zoledronic acid were given the same volume of saline (approximately 6 μL) intramuscularly every 4 weeks.

**Figure 1:**
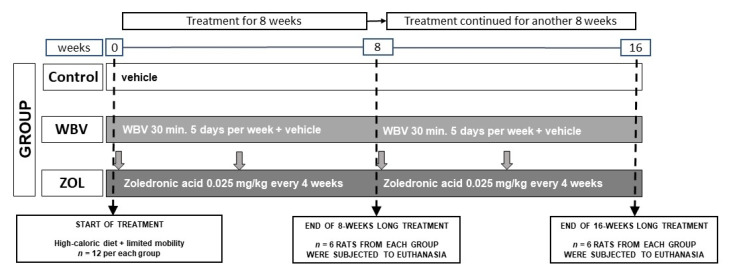
Scheme of the experimental design. Rat from all groups were fed a high-calorie diet and kept in limited mobility conditions. Rats from the WBV group were subjected to whole-body vibration treatment (30 min, 5 days per week), while rats from the ZOL group were injected intramuscularly with zoledronic acid at a dose of 0.025 mg/kg body weight every 4 weeks. Rats from the Control and WBV groups were injected with the vehicle (0.9% NaCl) at the same volume. Vertical gray arrows show the time of injections. At the end of the 8- or 16-week-long treatment, *n* = 6 animals from each group were euthanized.

The body weight of the rats was monitored weekly. The presence of obesity in each animal was determined using the Lee index, which is the ratio of the cubic root of body weight (g) to the rat’s nasal–anal length (cm) [40]. Rats with a Lee index ≥ 0.3 were classified as obese rats, and those with a Lee index < 0.3 were classified as normal-weight rats. No changes were observed at the injection site on the skin and subcutaneous tissue. After the 8th and 16th weeks, 6 animals from each group were anesthetized with an intraperitoneal injection of ketamine + xylazine solution in NaCl (100 mg/kg + 10 mg/kg), according to recommendations for anesthesia and euthanasia of experimental animals and were euthanized by cervical dislocation. Figure 1 shows the scheme of the study.

### 2.2. Bone Preparation and Histomorphometry Analysis

Right femora were isolated from rats, cleaned of any remaining soft tissue, and the distal sections of bone (including epiphysis, metaphysis, and part of the shaft) were fixed in buffered (pH 7.0) 4% formaldehyde for 24 h, and then were rinsed with running tap water. Bone tissue decalcification with decalcifier solution (Rapid Descaler, Kaltek S.r.l., Saonara (PD), Italy) was carried out for 48 h at room temperature. The decalcified material was washed with distilled water, dehydrated in graded EtOH solutions, and cleared with xylene. The resulting bone material was paraffin-fixed and cut into 5-μm-thick sections using a microtome (HM360, Microm, Walldorf, Germany). The sections were stained with Picro Sirus Red (PSR), observed, and photographed under a bright-field microscope to assess the basic histomorphometry of the trabecular bone in the distal metaphysis of the femur: relative bone volume (bone volume fraction), trabecular thickness, trabecular spacing, and fractal dimension of the trabeculae [41,42]. The percentage of immature collagen in PSR-stained sections observed under polarized light (seen as green) was calculated from pixel counting as a percentage of total collagen content [43]. Goldner trichrome stain was used to assess the growth plate morphometry. Microscopic images were collected using a light microscope (CX43, Olympus, Tokyo, Japan), and all analyses were performed using ImageJ 1.53 software (National Institutes of Health, Bethesda, MD, USA; accessible at: http://rsb.info.nih.gov/ij/index.html, accessed on 15 December 2021) [44].

### 2.3. Immunohistochemical Analysis

Following deparaffinization in xylene and rehydration with decreasing concentrations of ethanol followed by distilled water, immunohistochemical reactions were performed on the tissue sections according to a protocol described previously [45]. In brief, in order to reduce the non-specific background staining due to endogenous peroxidase, the slides were incubated in hydrogen peroxide (3% hydrogen peroxide in deionized water) for 10 min. The sections were then washed two times in a PBS buffer solution. The next step was to recover the antigenicity of the tested material by enzymatic reactions using the proteinase K (Sigma-Aldrich, St. Louis, MO, USA). This step took 10 min and was carried out at 37 °C. Sections were then washed twice in PBS buffer and incubated in pre-antibody blocking solution (Santa Cruz Biotechnology, Inc., Dallas, TX, USA) for 5 min at room temperature and washed twice with PBS. The sections were then incubated with the primary antibodies in a humidity chamber for 1 hour at room temperature. Rabbit polyclonal anti-osteoprotegerin (OPG; ab73400; Abcam, Cambridge, UK) and mouse monoclonal anti-receptor activator for nuclear factor kappa-B ligand (RANKL; ab239607; Abcam, Cambridge, UK) antibodies were diluted (1:100) in Diamond antibody diluent (Cell Marque Corp., Rocklin, CA, USA) and used as primary antibodies. Negative control sections for each antibody were obtained by identical immunohistochemical staining, except for the primary antibody. A PBS buffer was used instead of the primary antibody. The sections were then washed two times in PBS buffer. Post-antibody blocking solution (DPVB110HRP, BrightVision, Two-Step Detection System Goat Anti-Mouse/Rabbit HRP; Immunologic WellMed B.V., Duiven, Netherlands) was applied for 15 min, followed by two washes in PBS buffer. The sections were then incubated with PolyHRP-Goat Anti-Mouse/Rabbit IgG (DPVB110HRP, BrightVision, two-step detection system Goat Anti-Mouse/Rabbit HRP, Immunologic WellMed B.V., Duiven, Netherlands) for 30 min at room temperature. After the sections were washed twice in PBS buffer, they were developed in 3,3′-diaminobenzidine tetrahydrochloride (DAB D5905; Sigma-Aldrich, St. Louis, MO, USA), which was used as a chromogen, for 15 min at room temperature. Mayer haematoxylin (MHS32-1L; Sigma-Aldrich, St. Louis, MO, USA) was used for counterstain. The intensity of the immunoreaction was measured using a light microscopes (CX43 and BX63, Olympus, Tokyo, Japan) both by determining the percentage of cells with a positive reaction and by quantitatively comparing the average intensity of pixels on photomicrographs, which were first converted into negatives and then into 8-bit grayscale digital images, with a scale from 0 (white pixel) to 255 (black pixel), where the higher the pixel value, the higher the intensity of the immunohistochemical reaction [46,47]. For the immunoreactive cell counting procedure, four randomly selected areas of the trabecular bone were measured for each slide and the percentage of positive immunoreactive cells was reported. The intensity of immunoreactivity was measured in 12 randomly selected areas of positive signal in the trabecular bone and measured separately for cells (osteocytes) and bone matrix. All analyses were performed using ImageJ 1.53t software [44].

### 2.4. Statystical Anlysis

The statistical analysis was performed using GraphPad Prism 9 for Windows Version 9.4.1. The sample size was calculated for one-way ANOVA, an α of 0.05 and power at 0.8 [48]. Data in the literature indicate that the sample size of *n* = 6 has a power of 80% to detect a change of 6% in bone volume fraction, 11% in trabecular space, and 7% in trabecular thickness assuming a 5% significance level [49,50]. Normality was assessed using the Shapiro–Wilk test, while the homogeneity of the variance was studied using Levene’s test. A one-way ANOVA was used to assess the observed changes and post-hoc Tukey’s test was applied to evaluate the groups’ differences in the analyzed parameters. Non-parametric data were analyzed using a Kruskal–Wallis H test. For all tests, a *p*-value < 0.05 was established as statistically significant. All of the data were reported as mean ± standard deviation.

## 3. Results

At the beginning of the experiment, the mean rat body mass and the Lee index were 188 g and 0.283, 189 g and 0.285, 190 g and 0.285 in the Control, WBV, and ZOL group, respectively (*p* > 0.05), indicating that the rats in all groups did not show obesity (the Lee index < 0.3). After 8 weeks of the high-caloric diet irrespective of other treatment, all rats gained weight at similar rate (the mean body weight was 279 g, 284 g, and 293 g in the Control, WBV and ZOL group, respectively; *p* > 0.05) and the Lee index indicated the appearance of obesity in all groups (0.304, 0.307, 0.310, in the Control, WBV and ZOL group, respectively; *p* > 0.05). At the end of the 16th week of treatment, the obesity in the rats persisted (0.308, 0.310, and 0.310 in the Control, WBV, and ZOL group, respectively), but still no difference between the groups was observed (*p* < 0.05).

### Histomorphometry

**Figure 2:**
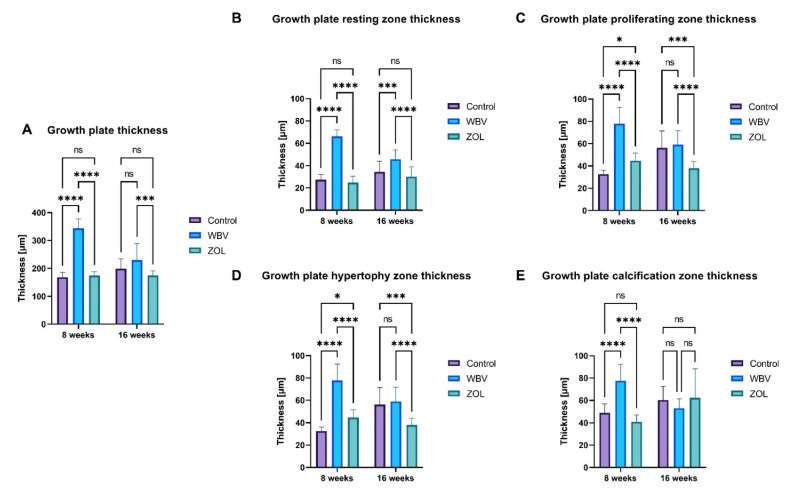
The effect of whole-body vibration (WBV) or zoledronic acid (ZOL) supplementation on the thickness of zones in the growth plate cartilage in rats subjected to obesity and immobility: (**A**) total growth plate thickness; (**B**) resting zone; (**C**) proliferating zone; (**D**) hypertrophy zone; (**E**) calcification zone. Control—the control group included rats subjected to obesity and immobility not treated with WBV or ZOL; WBV—the WBV group included rats subjected to obesity and immobility with whole-body vibration treatment (30 min, 5 days per week); ZOL—the ZOL group included rats subjected to obesity and immobility and injected intramuscularly with zoledronic acid at a dose of 0.025 mg/kg body weight every 4 weeks. Data are mean values ±SD from *n* = 6 rats. Statistical significance: ns—not significant; * *p* < 0.05; *** *p* < 0.001; **** *p* < 0.0001 (Tukey’s HSD test or Kruskal–Wallis H test).

The thicker growth plate was noted in the WBV group compared to the Control and ZOL groups after 8 weeks (Figure 2A); after 16 weeks, rats from the WBV group still had the thicker growth plate, but in comparison only to the ZOL group. The thickness of the resting, proliferating, hypertrophic, and calcification zones after 8 weeks also was significantly higher compared to the Control and ZOL group (Figure 2B–E). Moreover, the proliferating and hypertrophic zones were significantly thicker after 8 weeks in the ZOL group compared to corresponding zones in the Control group. The thickness of resting zone after 16 weeks in the WBV group was significantly higher compared to the values in the Control and ZOL groups. The thickness of the proliferating and hypertrophy zones was significantly higher in the Control and WBV groups after 16 weeks compared to the thickness in the ZOL group (Figure 2C,D). Other changes were not observed.

**Figure 3:**
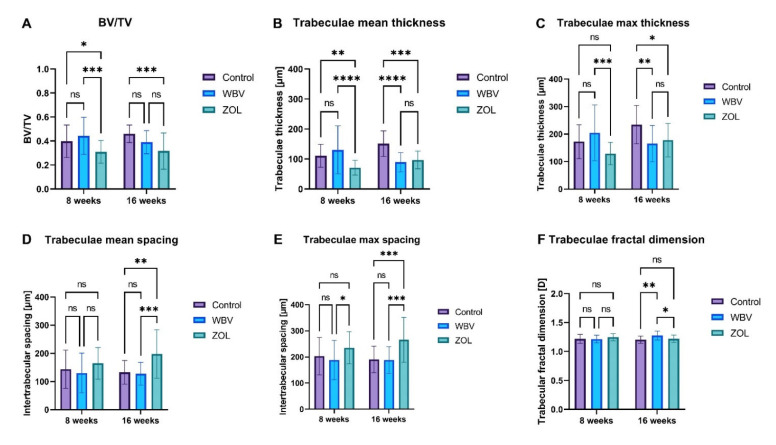
The effect of whole-body vibration (WBV) or zoledronic acid (ZOL) supplementation on the trabecular morphology in femur distal metaphysis in rats subjected to obesity and immobility: (**A**) bone volume fraction (BV/TV); (**B**) trabeculae maximum thickness; (**C**) trabeculae mean thickness; (**D**) trabeculae mean spacing; (**E**) trabeculae maximum spacing; (**F**) trabeculae fractal dimension. Control—the control group included rats subjected to obesity and immobility not treated with WBV or ZOL; WBV—the WBV group included rats subjected to obesity and immobility with whole-body vibration treatment (30 min, 5 days per week); ZOL—the ZOL group included rats subjected to obesity and immobility and intramuscularly with zoledronic acid at a dose of 0.025 mg/kg body weight every 4 weeks. Data are mean values ± SD from *n* = 6 rats. Statistical significance: ns—not significant; * *p* < 0.05; ** *p* < 0.01; *** *p* < 0.001; **** *p* < 0.0001 (Tukey’s HSD test or Kruskal–Wallis H test).

The histomorphology of trabecular bone is presented in Figure 3. The values of the mean thickness and BV/TV were higher in the WBV group compared to the value noted in the ZOL group, which was also lower compared to the value of the mean thickness in the Control group (Figure 3A). The maximum thickness of trabeculae after 8 weeks was significantly higher compared to the thickness only in the ZOL group (Figure 3C). Trabeculae maximum spacing was higher in the ZOL group compared to the spacing in the WBV group (Figure 3E).

The mean and maximum thickness of trabeculae after 16 weeks was significantly higher in the Control group compared to the thickness noted in the WBV and ZOL group (Figure 3B,C). However, the maximum and mean spacing were significantly higher in the ZOL group compared to the spacing noted in the Control and WBV groups. The value of BV/TV noted in the Control group differs only from the value noted in the ZOL group (Figure 3A). The fractal dimension was significantly higher in the WBV group compared to the value noted in the other groups (Figure 3F). Other changes were not noted.

**Figure 4:**
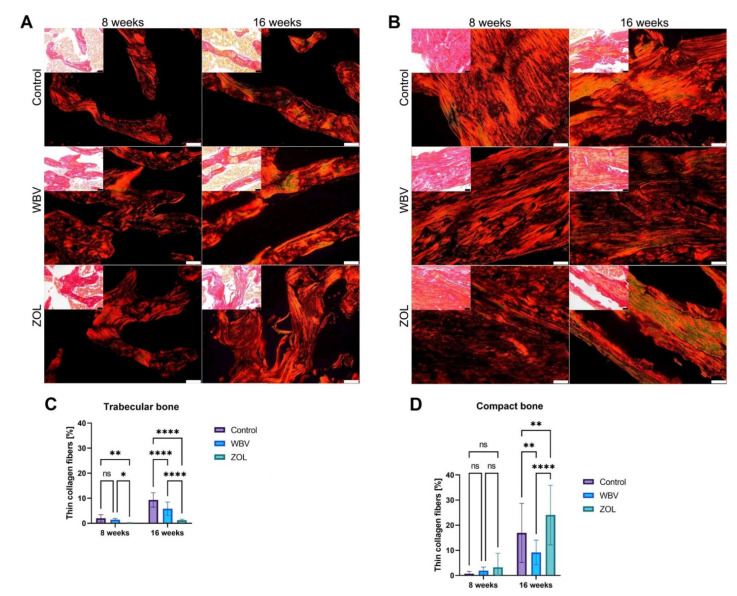
Thin (immature) collagen content in femora trabeculae in rats subjected to obesity and immobility: representative images of PRS-stained sections of (**A**) trabecular and (**B**) compact bone observed in polarized light (big images) and bright light (small images). The thin, immature collagen fibers are green and the mature fibers are orange or red. Insets: the same sections observed in bright-field. All scale bars represent 50 μm; bar graphs of thin (immature) collagen content in (**C**) femora trabeculae and (**D**) femora compact bone. Data are mean values ± SD from *n* = 6 rats. Control—the control group included rats subjected to obesity and immobility not treated with WBV or ZOL; WBV—the WBV group included rats subjected to obesity and immobility with whole-body vibration treatment (30 min, 5 days per week); ZOL—the ZOL group included rats subjected to obesity and immobility and injected intramuscularly with zoledronic acid at a dose of 0.025 mg/kg body weight every 4 weeks. Statistical significance: ns—not significant; * *p* < 0.05; ** *p* <0.01; **** *p* < 0.0001 (Tukey’s HSD test or Kruskal–Wallis H test).

Immature collagen content is presented in Figure 4. Representative images of PSR- stained sections of trabecular and compact bone are presented in polarized light (Figure 4A,B, respectively; big images). The content of immature collagen in the trabecular bone was the lowest in the ZOL group after 8 weeks compared to other groups (Figure 4C). After 16 weeks of treatment, the rats in the Control group had the highest content of immature collagen in their trabecular bone. Lower content was noted in the WBV group, and the lowest in the ZOL group; while the content of immature collagen in compact bone was the highest in the ZOL group, lower in the Control group, and the lowest in the WBV group (Figure 4C). For compact bone, the differences in immature collagen content were observed only after the 16-week-long treatment, with the lowest content observed in the WBV group and the highest in the ZOL group. Other changes were not noted.

**Figure 5:**
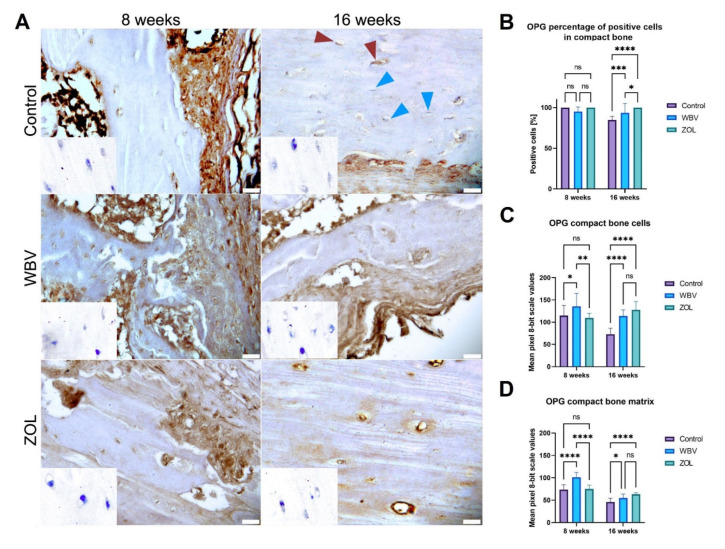
The immunohistochemical reaction for osteoprotegerin (OPG) in the compact bone of femora of rats subjected to obesity and immobility: (**A**) Representative images of the immunohistochemical reactions for OPG. Brown and blue arrowheads indicate examples of positive and negative reaction cells, respectively. Insets show control immunohistochemical staining without primary antibody. All scale bars represent 50 μm; bar graphs show the (**B**) the percentage of OPG-positive cells and the intensity of immunoexpression of OPG (**C**) in cells (osteocytes), and (**D**) compact bone matrix measured by the 8-bit grayscale pixel brightness value (the higher the pixel value, the higher the intensity of the immunohistochemical reaction). Data are mean values ± SD from *n* = 6 rats. Control—the control group included rats subjected to obesity and immobility not treated with WBV or ZOL; WBV—the WBV group included rats subjected to obesity and immobility with whole-body vibration treatment (30 min, 5 days per week); ZOL—the ZOL group included rats subjected to obesity and immobility and injected intramuscularly with zoledronic acid at a dose of 0.025 mg/kg body weight every 4 weeks. Statistical significance: ns—not significant; * *p* < 0.05; ** *p* < 0.01; *** *p* < 0.001; **** *p* < 0.0001 (Tukey’s HSD test or Kruskal–Wallis H test).

The representative images of the immunohistochemical reactions for OPG in the compact bone are presented in Figure 5A. The number of the OPG-positive cells in the compact bone after 16 weeks was the highest in the ZOL group, lower in the WBV group, and the lowest in the Control group (Figure 5B). The most intense immunoreaction in the bone cells was observed in the WBV group after 8 weeks. The same result was observed for the intensity of the OPG immunoreaction in the matrix. After 16 weeks, the most intensive OPG immunoreaction in the bone cells was observed in the ZOL and WBV groups compared to the intensity in the Control group. The intensity of the OPG immunoreaction in the matrix of the compact bone was lower in the Control group compared to the intensity observed in the WBV and ZOL groups. Other changes were not noted.

**Figure 6:**
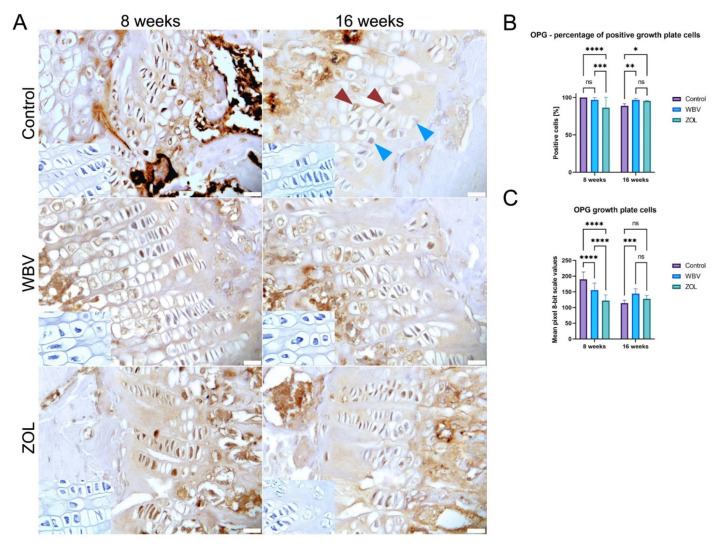
The immunohistochemical reaction for osteoprotegerin (OPG) in the growth plate of femora of rats subjected to obesity and immobility: (**A**) Representative images of the immunohistochemical reactions for OPG. Brown and blue arrowheads indicate examples of positive and negative reaction cells, respectively. Insets show control immunohistochemical staining without primary antibody. All scale bars represent 50 μm; bar graphs show (**B**) percentage of cells showing positive reaction to OPG, and (**C**) the intensity of immunoexpression of OPG in the growth plate cells measured by the 8-bit grayscale pixel brightness value (the higher the pixel value, the higher the intensity of the immunohistochemical reaction). Data are mean values ± SD from *n* = 6 rats. Control—the control group included rats subjected to obesity and immobility not treated with WBV or ZOL; WBV—the WBV group included rats subjected to obesity and immobility with whole-body vibration treatment (30 min, 5 days per week); ZOL —the ZOL group included rats subjected to obesity and immobility and injected intramuscularly with zoledronic acid at a dose of 0.025 mg/kg body weight every 4 weeks. Statistical significance: ns—not significant; * *p* < 0.05; ** *p* < 0.01; *** *p* < 0.001; **** *p* < 0.0001 (Tukey’s HSD test or Kruskal–Wallis H test).

The representative images of the immunohistochemical reactions for OPG in the growth plate are presented in Figure 6A. The percent of positive OPG in the growth plate cells is presented in Figure 6B. The lowest number of OPG-positive cells in the growth plate was in the ZOL group after 8 weeks compared to other groups, while after 16 weeks the opposite situation was observed because the number of OPG-positive cells in the Control group was the lowest. After the 8-week-long treatment, the lowest intensity of the OPG reaction was observed in the ZOL group, followed by the WBV groups, and the highest intensity was noted in the Control group. In turn, after 16 weeks, the lowest intensity was in the Control and the ZOL groups, and the intensity of the OPG reaction in the WBV group was significantly higher when compared to the Control group. Other changes were not noted.

**Figure 7:**
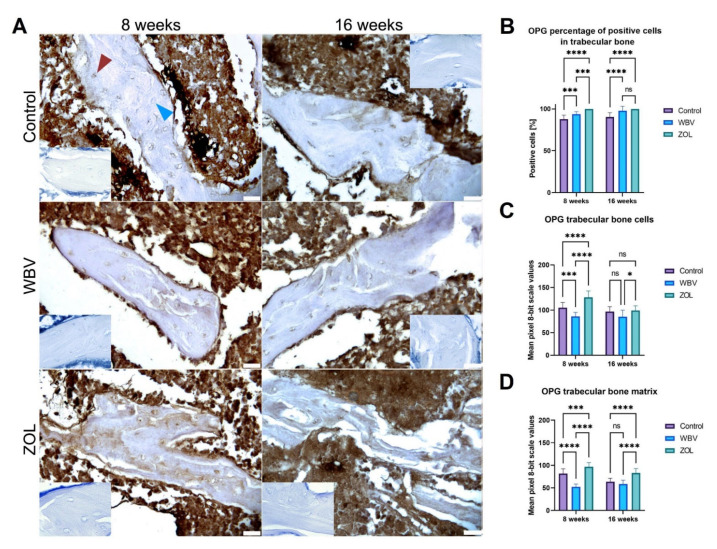
The immunohistochemical reaction for osteoprotegerin (OPG) in the trabeculae of distal metaphysis of femora of rats subjected to obesity and immobility: (**A**) Representative images of the immunohistochemical reactions for OPG. Brown and blue arrowheads indicate examples of positive and negative reaction cells, respectively. Insets show control immunohistochemical staining without primary antibody. All scale bars represent 50 μm; bar graphs show the (**B**) the percentage of OPG-positive cells and the intensity of immunoexpression of OPG (**C**) in trabecular bone cells, and (**D**) trabecular bone matrix measured by the 8-bit grayscale pixel brightness value (the higher the pixel value, the higher the intensity of the immunohistochemical reaction). Data are mean values ± SD from *n* = 6 rats. Control—the control group included rats subjected to obesity and immobility not treated with WBV or ZOL; WBV—the WBV group included rats subjected to obesity and immobility with whole-body vibration treatment (30 min, 5 days per week); ZOL—the ZOL group included rats subjected to obesity and immobility and injected intramuscularly with zoledronic acid at a dose of 0.025 mg/kg body weight every 4 weeks. Statistical significance: ns—not significant; * *p* < 0.05; *** *p* < 0.001; **** *p* < 0.0001 (Tukey’s HSD test or Kruskal–Wallis H test).

The OPG immunoreaction in trabecular bone is presented in Figure 7. The representative images are presented in Figure 7A. The highest number of OPG-positive cells in the trabecular bone was in the ZOL group after 8 weeks, lower in the WBV group, and the lowest in the Control group; after 16 weeks, the difference was noted in OPG-positive cells between the Control group and the ZOL and WBV groups (Figure 7B). The lowest intensity of the OPG reaction both in the trabecular bone cells and trabecular bone matrix was observed after 8 weeks in the WBV group, followed by the Control group, and the highest in the ZOL group (Figure 7C,D). In the case of 16-week-long treatment, the intensity of the OPG immunoreaction in the bone cells was lower in the WBV group when compared to the ZOL group. (Figure 7C). In the bone matrix, the intensity of the OPG reaction after 16 weeks in the Control and WBV groups did not differ, and both were significantly lower than that observed in the ZOL group (Figure 7D). Other changes were not noted.

**Figure 8:**
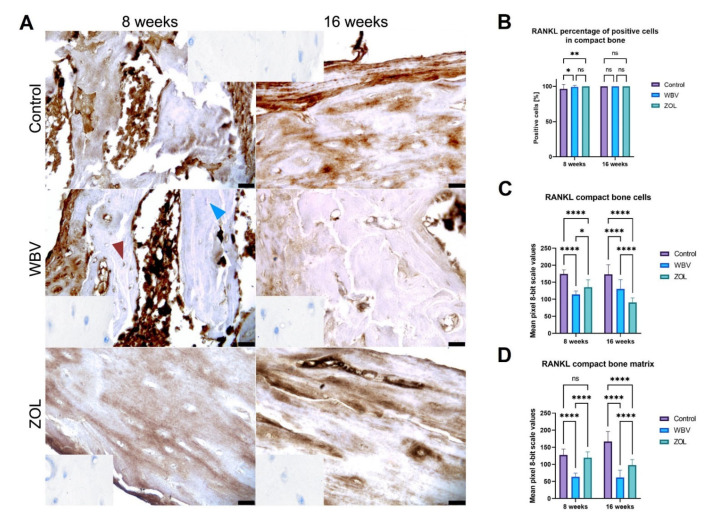
The immunohistochemical reaction for receptor activator for nuclear factor kappa-B ligand (RANKL) in the compact bone of femora of rats subjected to obesity and immobility: (**A**) Representative images of the immunohistochemical reactions for RANKL. Brown and blue arrowheads indicate examples of positive and negative reaction cells, respectively. Insets show control immunohistochemical staining without primary antibody. All scale bars represent 50 μm; bar graphs show the (**B**) the percentage of RANKL-positive cells and the intensity of immunoexpression of RANKL (**C**) in cells (osteocytes), and (**D**) compact bone matrix measured by the 8-bit grayscale pixel brightness value (the higher the pixel value, the higher the intensity of the immunohistochemical reaction). Data are mean values ± SD (whiskers) from *n* = 6 rats. Control—the control group included rats subjected to obesity and immobility not treated with WBV or ZOL; WBV—the WBV group included rats subjected to obesity and immobility with whole-body vibration treatment (30 min, 5 days per week); ZOL—the ZOL group included rats subjected to obesity and immobility and injected intramuscularly with zoledronic acid at a dose of 0.025 mg/kg body weight every 4 weeks. Statistical significance: ns—not significant; * *p* < 0.05; ** *p* < 0.01; **** *p* < 0.0001 (Tukey’s HSD test or Kruskal–Wallis H test).

The intensity of the RANKL immunoreaction in compact bone is presented in Figure 8, with representative images presented in Figure 8A. The lowest number of RANKL- positive cells in the compact bone was in the Control group after 8 weeks compared to the other groups; after 16 weeks, no differences between groups were noted (Figure 8B). The most intense RANKL immunoreaction in the bone cells of the compact bone was observed in the Control group after 8 weeks, less intensity was noted in the ZOL group, and the lowest intense reaction was observed in the WBV group (Figure 8C). The most intense RANKL immunoreaction in the bone cells of the compact bone was observed in the Control group after the 16-week-long treatment as well. Less intensity of the RANKL immunoreaction in the bone cells was noted in the WBV group and the lowest intensity reaction was observed in the ZOL group. The intensity of the RANKL immunoreaction in the matrix was lower in the WBV group compared to the intensity noted in other groups (Figure 8D), while the intensity of the RANKL immunoreaction in the matrix after 8 weeks was the highest in the Control group, lower intensity was noted in the ZOL group, and the lowest in the WBV group. Other changes were not noted.

**Figure 9:**
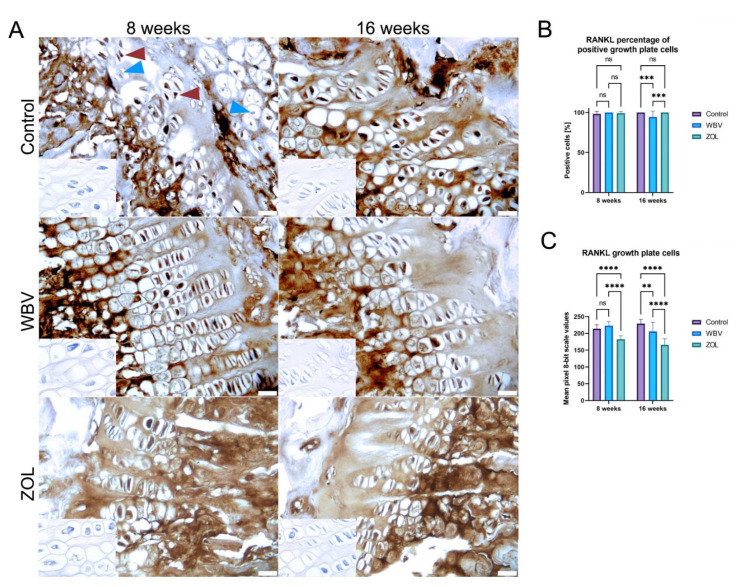
The immunohistochemical reaction for receptor activator for nuclear factor kappa-B ligand (RANKL) in the growth plate of femora of rats subjected to obesity and immobility: (**A**) Representative images of the immunohistochemical reactions for RANKL. Brown and blue arrowheads indicate examples of positive and negative reaction cells, respectively. Insets show control immunohistochemical staining without primary antibody. All scale bars represent 50 μm; bar graphs show (**B**) percentage of cells showing positive reaction to RANKL, and (**C**) the intensity of immunoexpression of RANKL in the growth plate cells measured by the 8-bit grayscale pixel brightness value (the higher the pixel value, the higher the intensity of the immunohistochemical reaction). Data are mean values ± SD from *n* = 6 rats. Control—the control group included rats subjected to obesity and immobility not treated with WBV or ZOL; WBV—the WBV group included rats subjected to obesity and immobility with whole-body vibration treatment (30 min, 5 days per week); ZOL—the ZOL group included rats subjected to obesity and immobility and injected intramuscularly with zoledronic acid at a dose of 0.025 mg/kg body weight every 4 weeks. Statistical significance: ns—not significant; ** *p* < 0.01; *** *p* < 0.001; **** *p* < 0.0001 (Tukey’s HSD test or Kruskal–Wallis H test).

The representative images of the observed RANKL immunoreaction in the growth plate are presented in Figure 7B, the percent of positive RANKL in the growth plate cells is presented in Figure 9B. The lowest number of RANKL-positive cells in the growth plate was noted in the WBV group after 16 weeks compared to other groups, a higher percentage was noted in the ZOL group, and the highest in the Control group. Other changes were not noted. The lowest intensity of RANKL immunoreaction after 8 weeks was noted in the ZOL group, which was significantly lower than in the other two groups (Figure 9C). After 16 weeks, the lowest intensity in RANKL immunoreaction was still observed in the ZOL group, followed by the WBV group, and the highest observed in the Control group (Figure 9C).

**Figure 10:**
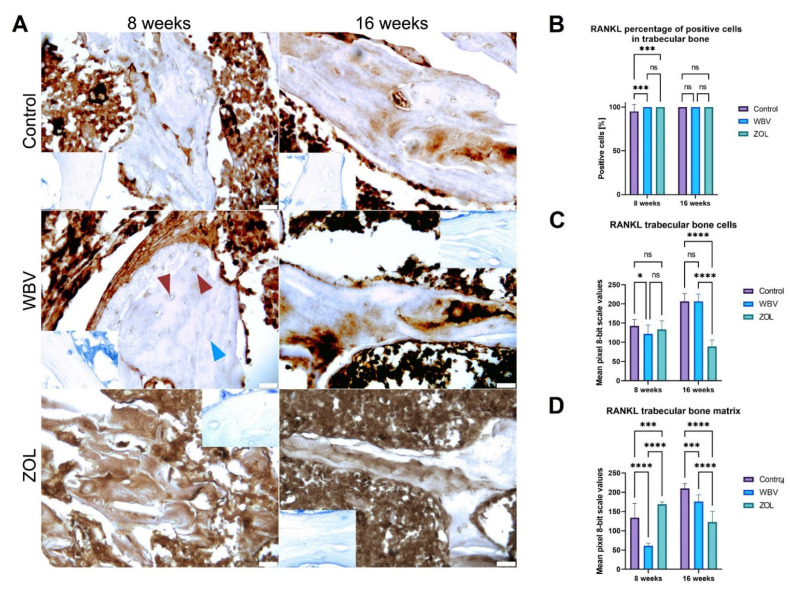
The immunohistochemical reaction for receptor activator for nuclear factor kappa-B ligand (RANKL) in the trabeculae of distal metaphysis of femora of rats subjected to obesity and immobility: (**A**) Representative images of the immunohistochemical reactions for RANKL. Brown and blue arrowheads indicate examples of positive and negative reaction cells, respectively. Insets show control immunohistochemical staining without primary antibody. All scale bars represent 50 μm; bar graphs show the (**B**) the percentage of RANKL-positive cells and the intensity of immunoexpression of RANKL (**C**) in trabecular bone cells, and (**D**) trabecular bone matrix measured by the 8-bit grayscale pixel brightness value (the higher the pixel value, the higher the intensity of the immunohistochemical reaction). Data are mean values ± SD from *n* = 6 rats. Control—the control group included rats subjected to obesity and immobility not treated with WBV or ZOL; WBV—the WBV group included rats subjected to obesity and immobility with body vibration treatment (30 min, 5 days per week); ZOL—the ZOL group included rats subjected to obesity and immobility and injected intramuscularly with zoledronic acid at a dose of 0.025 mg/kg body weight every 4 weeks. S Statistical significance: ns—not significant; * *p* < 0.05; *** *p* < 0.001; **** *p* < 0.0001 (Tukey’s HSD test or Kruskal–Wallis H test).

The representative images of the RANKL immunoreaction in trabecular bone are presented in Figure 10A. The lowest number of RANKL-positive cells in trabecular and compact bone was in the Control group after the 8-week-long treatment compared to the other groups; after 16 weeks, no differences between the groups were noted (Figure 10B). The most intense RANKL immunoreaction in the bone cells was observed in the Control group after 8 weeks compared to the intensity noted in the WBV group (Figure 10C). The intensity of the RANKL immunoreaction in the matrix was the most intense in the ZOL group, less intense in the Control group, and the lowest intensity was noted in the WBV after 8 weeks (Figure 10D). After 16 weeks, the most intensive RANKL immunoreaction was observed in the bone cells in the Control and WBV groups compared to the intensity in the ZOL group (Figure 10C). The intensity of the RANKL immunoreaction in the matrix of the trabeculae was the highest in the Control group, lower in the WBV group, and the lowest in the ZOL group (Figure 10D). Other changes were not noted.

## 4. Discussion

Obesity is a chronic condition that is a major health problem, the prevalence of which is increasing worldwide due to an aging population, increasing incidence of obesity, and lifestyle changes. Even in the case of obesity, there is a state in which osteoporosis coexists as a condition with low bone mass and degradation of bone microarchitecture, and sarcopenia, in which a loss of muscle mass, strength, and function is observed [1,2]. In general, obesity and immobility significantly increase the risk of fractures and entails significant socioeconomic costs. A better understanding of the interaction between mechanical stress and osteoporosis may contribute to the development of new therapeutic agents that would affect the musculoskeletal system as a whole. Therefore, due to the non-specificity of symptoms, osteopenia or osteoporosis associated with obesity remains largely undiagnosed.

Studies confirm that long-term consumption of diets high in fat and sugar have a negative effect on bone formation. As evidence, Zernicke et al. [51] reported that rodents fed on a combined high-fat, high-sucrose diet for 24 months had reduced cortical bone thickness and strength compared to those fed on a low-fat complex carbohydrate diet. Bass et al. [52] reported that a low-fat/high-glucose diet impaired bone quality, suggesting that sucrose may impair bone remodeling. Another study reported that fractures in postmenopausal women and in older obese men were significantly higher than in the general population, although risk factors were common in both groups [6]. Chen et al. [52] described no effect of a high-fat diet on tibial BMD in young female rats. However, at the same time, the strength of the lumbar region and femoral neck worsened, probably due to a decrease in the cortical shell region, which was observed in comparison with the standard diet. Other authors reported that in young male mice C57BL/6, there were no significant changes in the characteristics of bone tissue of the compact layer and its strength after consuming a high-calorie diet for a long time [53,54]. From these studies, it is evident that hypodynamia and obesity can influence the pathogenesis and loss of bone tissue, similar to what is observed in sex hormone deficiency [37].

Using a model with a high-calorie diet and limited mobility, we showed that starting from the 8th week of the study, the histomorphology of the distal epiphysis and metaphysis of the femur was disturbed. Vibration had no statistically significant effect on cancellous bone volume fraction and trabecular spacing, and ZOL partially reduced the thickness of bone trabeculae at week 16, as evidenced by decreased trabecular thickness and increased trabecular spacing. This confirms that antiresorptive therapy alone is not sufficient to treat/prevent osteopenia in individuals with sarcopenia. In particular, in a study on humans, it was found that obese women had an increased risk of fractures compared to persons who do not suffer from it. Insufficient effectiveness of antiresorptive therapy was also noted in the sample [55]. In other studies on rats, it has been established that as weight increases in animals consuming a high-fat diet [56], the volume of cancellous bone decreases. One possible explanation for this is that obesity may reduce the osteogenic capacity of mesenchymal stem cells obtained from bone marrow, as demonstrated in vitro [57].

From the graphs in Figure 3, it is obvious that long-term WBV did not improve bone morphology at week 16. However, short-term treatment with WBV (week 8) resulted in an increase in mean trabecular thickness, but the results were statistically insignificant. We have previously established that a model of obesity/immobility-related osteoporosis is able to induce similar bone mineral deficiencies in the proximal femur of young male rats compared to controls on a standard low-fat, low-sugar diet [58] and, in this case, vibration and zoledronic acid improved the crystalline structure of the bones.

Given the complex structure of the bone, we assume that such indicators are not sufficient for establishing a diagnosis of osteoporosis and predicting fractures. Fractal geometry is often used to describe irregular porous media, which are characterized by a non-Euclidean fractal dimension, and the fractal dimension is a non-integer. This is probably explained by the fact that the fractal dimension reflects changes in the bone microstructure [59]. For osteoporosis, a larger fractal dimension of the bone matrix indicates smaller bone loss (smaller porosity), while a smaller fractal dimension of the bone matrix indicates greater bone loss (or larger porosity). The results show that vibration of 0.3 g, 50 Hz improves the bone condition in the long term, but it also becomes evident that a serious drawback of our study is its term and limitations regarding the relatively young age of the rats.

Bone is a dynamic organ that continuously undergoes significant changes—remodeling, which includes bone resorption by osteoclasts and bone formation by osteoblasts [60]. Therefore, bone mass at any particular moment in time reflects the balance between bone formation and bone resorption. Osteoblasts have been shown to regulate osteoclast involvement and activity through the expression of the receptor activator of NF-B ligand (RANKL) and osteoprotegerin (OPG). The intensity of the immunohistochemical reaction of RANKL and OPG in the bone cells in the research groups also showed some differences. Increased intensity of RANKL expression leads to increased osteoclastic activity and bone resorption in a sarcopenia model. Zoledronic acid induced a decrease in the intensity of the RANKL immunoreaction in the trabecular, compact, and germinal plate cells, which was especially marked at the 16th week. However, vibration had a positive effect on the intensity of RANKL expression in the trabecular and cortical bone matrix starting from the 8th week of the study, which indicates the activation of the remodeling processes. During this period, the number of OPG-positive cells in the trabecular bone also increased. However, in the compact layer, similar changes were observed only at the 16th week. Increased expression of osteoprotegerin cells may indicate prevention of RANK activation and may therefore reduce osteoclast differentiation and activation. The number of OPG-positive cells in the trabeculae also increased during the 8th–16th weeks of the experiment under the influence of vibration and ZOL, and in the germ layer such changes were observed only at the 16th week. Immunohistochemical reactions for OPG increased in the cells and matrix of the trabecular layer of the femur in the ZOL group compared to the results of the WBV and Control groups. However, the intensity of the immune reaction for OPG in the germ layer cells of the ZOL group was significantly lower than the results of the WBV group and the Control group. Perhaps such active changes in the activity of the bone cells in rats with obesity and exposure to vibration and bisphosphonate explain the slight thinning of trabeculae during the experiment, while improving the trabecular fractal dimension. The model in general can lead to adverse bone development in skeletally mature male rats. In contrast, the remodeling processes of the RANKL/RANK/OPG system were detected and somewhat stabilized in animals with additional use of vibration. In conclusion, the administration of zoledronic acid restores cancellous bone tissue without affecting the germinal plate of the distal femur in rodents.

An important factor for the estimation of bone tissue is the assessment of its mineral component. However, its strength is determined not only by BMD values, but by the combination of amorphous (collagen fibers) and mineral (hydroxyapatite and calcite) components determining its parameters, such as crack generation and propagation, strength, stiffness, toughness, elastic constants, deformation, stress, strain, etc. [18]. To detect collagen fibers, we used Picrosirius red (PSR) staining, which enabled the establishment of its ratio in the trabecular and cortical layers in the Control and experimental groups. Obesity with immobility led to an increase in the level of collagen fibers at the 16th week of the experiment, while this indicator decreased in the rats in the WBV and ZOL groups, probably due to the percentage increase in the mineral component, which was described in a previous study [58]. In the compact layer, zoledronic acid led to an increase in its amount both compared with the control and the WBV group, where the index was two-fold lower. The Control rats had less thinned femoral trabeculae compared with the WBV-treated rats. Thus, the impact of general vibration with an acceleration level of 3 m/s^2^ and a frequency of 50 Hz results in the activation of remodeling, and the changes occurring in the bone depend not only on the strength and nature of vibration impact, but also on the duration of its action.

The model of obesity/immobility-related osteoporosis throughout 16 weeks significantly affects the germinal plate condition in the distal part of the femur. The influence of vibration is accompanied by an increase in the thickness of the germinal plate, which was most pronounced in rats in the 8th week of the experiment. Zoledronic acid did not affect the thickness of the germinal plate layers, and the quantitative values were equal to the results found in the Control group.

Although diet and lifestyle are only a part of the many factors that affect bone mass, they are of particular importance for bone remodeling. According to the data, a high-calorie diet/restricted mobility appears to be harmful to the development of the trabecular bone layer, while the effect on the cortical plate is less intense. In the cortical layer, the intensity of the immune reaction of the OPG cells increased under the influence of vibration at the 8th week, and under the influence of ZOL it did so more at the 16th week. The OPG compact bone matrix also changed similarly under the influence of vibration at the 8th week and at the 16th week under the influence of ZOL. Furthermore, a decrease in the immunohistochemical reactions for the RANKL compact bone cells and matrix was observed at the 8th week. The decrease in thin collagen fibers under the influence of vibration probably indicated the activation of remodeling in the cortical layer, which was confirmed by the increase in the number of OPG-positive cells.

We compared our results with data in the literature, in which the authors, including McGee-Lawrence et al. [60], subjected wild-type male mice and leptin receptor-deficient (db/db) mice to daily treadmill exercise/WBV. Both methods reduced the body weight of the mice along with a decrease in lipid content and elevation in the level of circulating osteocalcin, which indicated an increase in the activity of osteoblasts in many areas of the skeleton [59]. Others tested the beneficial effects of whole-body vibration (WBV) training on physical condition and body mass in obese mice on high-fat diet. The rats under study were exposed to WBV with a low level of vibration acceleration (0.13 g) and high-intensity vibration (0.68 g) [61]. We noticed a reduction in body weight and normalization of biochemical parameters in obese mice and hypothesized that WBV may be an effective non-pharmacological method for health promotion and prevention of obesity induced by a high-calorie diet. It should be noted that an interesting fact in our experiments was that the trabecular areas of the femora in the WBV group were somewhat thinner compared to the Control, probably due to metabolism acceleration in the skeleton. However, it has been proven that the mineral density of bone tissue changes depending on the frequency of the vibration stimulus [62]. In addition, this increase indicates a possible osteogenic effect on mechanical oscillations of 10 and 20 Hz. Equivalent effects on BMD were observed in women in their postmenopausal period during physical exercises (pilates) [63].

Despite the fact that almost the only effective method of treatment and prevention of obesity/immobility-related osteoporosis is the normalization of body weight and physical activity, there are data on the use of classical antiresorptive therapy, the results of which are substantially described in the literature. Liu et al. [64] studied the safety and efficacy of zoledronic acid (single injection 5 mg, intravenous) as a potent antiresorptive bisphosphonate on bone remodeling condition in obese patients after bariatric surgery. The introduction of bisphosphonate can reduce the loss of BMD in the trabecular region of the spine compared to the control group [64].

High efficacy of bisphosphonates in the treatment of bone metastases, hypercalcemia, and osteoporosis is presented in the literature [25]. However, their influence may be associated with osteonecrosis of the jaw (ON). The authors observed a thirty-fold increase in the risk of developing osteonecrosis of the jaw after a course of zoledronic acid treatment in patients who smoked and were obese. Others investigated the effect of zoledronic acid on bone strength in ovariectomized (OVX) rodents. The OVX rats were administered a single intravenous injection of ZOL (20 μg/kg) and placed on a treadmill (15 m/min, 60 min/day, 5 days/week) for 12 weeks [65]. The zoledronic acid prevented trabecular bone loss induced by ovariectomy and its subsequent microarchitectural deterioration. It was shown that running on a treadmill to maintain bone strength and induce changes in bone metabolism was beneficial for bone formation. However, the combined effects of zoledronic acid and exercise applied simultaneously did not produce any synergistic or additive effects. Similar results were obtained by Hoffmann et al., 2017 [66], who compared the effects of vibration (WBV 70 Hz for 15 min) with osteoanabolic parathyroid hormone (PTH 40 μg/kg body weight/day) and mainly anti-resorptive strontium ranelate (SR 600 mg/kg of body weight/day). The authors revealed that PTH produced statistically significant improvements in biomechanical and structural properties, including bone mineral density (BMD) and trabecular bone quality. In contrast, WBV alone or combined therapy did not provide significant improvements [66].

Therefore, the obtained results enabled us to make the hypothesis that a high-calorie diet with limited mobility leads to a violation of the histomorphological structure of the femur. However, additional mechanical stimulation improves the condition of the trabecular layer in the early stages without additional use of bisphosphonates. In addition, using light microscopy, we recorded structural changes in the growth plate, in particular, disorganized cartilaginous zones and a decrease in the expression and number of OPG-positive cells, indicating that zoledronic acid when used systemically has a negative effect on cell proliferation, starting early (8th week). Although investigations of obesity/immobility-related osteoporosis models established a negative effect of obesity on bone metabolism, studies on humans are still controversial. Human obesity is a complex problem that generally involves excessive consumption of other nutrients, such as protein and minerals, which are known to affect bone metabolism. Conclusions about the effects of obesity on bone health in humans have been based on statistical correlations or modeling rather than randomized controlled trials. Therefore, controlled studies on obese/immobilized animal models are useful for elucidating the mechanisms by which both obesity and immobility affects bone metabolism.

## 5. Conclusions

A high-calorie diet in combination with a sedentary lifestyle leads to adverse femoral development in skeletally mature male rats, despite the potential impact of mechanical load caused by increased body weight. Zoledronic acid causes stabilization in remodeling processes and, accordingly, preservation of bone mass. In contrast, in animals with additional use of vibration, slightly reduced structural parameters of bones and the activation of remodeling were more pronounced in the early stages. Based on these assumptions, we hypothesized that the combined effects of exercise have beneficial effects on bone metabolism and may be beneficial in overweight individuals. Furthermore, short-term, high-frequency mechanical vibrations of the whole body can be used as an additional method of prevention/treatment of osteopenia.

## Data Availability

The data presented in this study are available on request from the corresponding authors.
